# Machine Learning in Vascular Medicine: Optimizing Clinical Strategies for Peripheral Artery Disease

**DOI:** 10.1007/s12170-024-00752-7

**Published:** 2024-11-04

**Authors:** Sean Perez, Sneha Thandra, Ines Mellah, Laura Kraemer, Elsie Ross

**Affiliations:** 1 Department of Surgery, University of California San Diego Health, La Jolla, San Diego, CA USA; 2 University of California San Diego School of Medicine, La Jolla, San Diego, CA USA; 3 General Surgery Department, Naval Medical Center San Diego, San Diego, CA USA; 4 Department of Surgery, Division of Vascular and Endovascular Surgery, University of California San Diego Health, 9300 Campus Point Drive #7403, La Jolla, San Diego, CA 92037 USA

**Keywords:** Artificial intelligence, Machine learning, Peripheral artery disease, Phenotyping, Risk stratification

## Abstract

**Purpose of Review:**

Peripheral Artery Disease (PAD), a condition affecting millions of patients, is often underdiagnosed due to a lack of symptoms in the early stages and management can be complex given differences in genetic and phenotypic characteristics. This review aims to provide readers with an update on the utility of machine learning (ML) in the management of PAD.

**Recent Findings:**

Recent research leveraging electronic health record (EHR) data and ML algorithms have demonstrated significant advances in the potential use of automated systems, namely artificial intelligence (AI), to accurately identify patients who might benefit from further PAD screening. Additionally, deep learning algorithms can be used on imaging data to assist in PAD diagnosis and automate clinical risk stratification.

ML models can predict major adverse cardiovascular events (MACE) and major adverse limb events (MALE) with considerable accuracy, with many studies also demonstrating the ability to more accurately risk stratify patients for deleterious outcomes after surgical intervention. These predictions can assist physicians in developing more patient-centric treatment plans and allow for earlier, more aggressive management of modifiable risk-factors in high-risk patients. The use of proteomic biomarkers in ML models offers a valuable addition to traditional screening and stratification paradigms, though clinical utility may be limited by cost and accessibility.

**Summary:**

The application of AI to the care of PAD patients may enable earlier diagnosis and more accurate risk stratification, leveraging readily available EHR and imaging data, and there is a burgeoning interest in incorporating biological data for further refinement. Thus, the promise of precision PAD care grows closer. Future research should focus on validating these models via real-world integration into clinical practice and prospective evaluation of the impact of this new care paradigm.

## Introduction

Peripheral artery disease (PAD), defined here, is a condition that reduces blood flow to the limbs due to narrowed arteries from plaque buildup and affects over 8 million people in the United States alone. While patients with PAD can experience leg pain with exercise (claudication), in the beginning stage it is often asymptomatic, which makes it difficult to diagnose and can delay treatment. If left untreated, the disease can advance to critical limb ischemia requiring amputation and is also associated with poor cardiovascular outcomes such as strokes, myocardial ischemia, and death. Risk factors for PAD include age > 50, Black race and chronic diseases such as diabetes, heart disease or kidney disease [1]. Early diagnosis and treatment of the disease is critical to improve quality of life and limb and cardiovascular outcomes.

Because PAD presents without noticeable symptoms in the early stage, it often remains underdiagnosed until later stages when symptoms arise. Some societies such as the American Heart Association (AHA) and American College of Cardiology (ACC) recommend that only populations at risk should be screened for the disease, while others such as the United States Preventative Task Force (USPSTF) recognize that there is a lack of evidence for screening at all in the asymptomatic patient population [2]. However, targeted screening leading to earlier diagnosis of PAD could allow physicians to more optimally medically manage patients, resulting in a larger impact on patient longevity and quality of life.

Machine learning (ML) is a branch of artificial intelligence (AI) which focuses on developing computer models that can “learn” from data and automate decision making with little human involvement. By analyzing large data sets, data scientists can develop computer-based models that calculate disease risk using complex mathematics and potentially make recommendations without needing intervention from humans. In recent years, ML and AI have been used in the fields of oncology, neurology, and many others to assist physicians in disease diagnosis and in clinical decision making [3] [4]. A benefit of ML for medical data analysis is that it can highlight non-linear relationships that might not otherwise be evident. Additionally, it exponentially increases efficiency by analyzing large data sets in short periods of time.

In this review paper we will discuss the use of ML in the screening, early diagnosis, and risk stratification of PAD. We begin by exploring how ML can analyze different data types commonly used to diagnose the condition such as the electronic health records (EHR), imaging and proteomics data. We will then discuss how ML can be leveraged for patient risk stratification. We will specifically review its use within the lens of outcome prediction, subtype clustering, and imaging.

### Early Diagnosis

The utilization of machine learning models for clinical decision support offers an opportunity to screen for and diagnose patients with PAD earlier in the disease process. PAD often has an atypical clinical presentation and can be clinically silent in patients with comorbidities or risk factors like diabetes. This clinical complexity may lead to disparities between the severity of stenosis and a patient’s symptom burden or functional capacity, thereby affecting management and interventional outcomes. ML approaches can identify predictive variables within these nonlinear relationships to identify PAD [5]. Current models have analyzed electronic health record data including clinical notes, proteomics, and imaging to optimize diagnosis.

#### Electronic Health Record Data

A wide array of data is stored in the EHR that can be utilized by ML algorithms to detect PAD status. This includes patient demographics, clinical risk factors like hypertension and coronary artery disease (CAD), physiological variables like blood pressure and ankle brachial index (ABI) values, and medications. Sonderman et al. combined these data including blood pressure, pulse pressure, history of smoking, and total cholesterol to high-density lipoprotein cholesterol ratio to assess individualized risk for PAD using an ML model and logistic regression (LR) techniques [6]. Their goal was to develop a model that could identify patients who should be screened for PAD with an ABI test. Using data from 1,089 patients, their model achieved 64% accuracy and an area under the curve (AUC), a measure of model discrimination, of 0.68 in a validation cohort [6]. Compared to age-based ABI screening alone, which achieved an AUC of 0.62, their model demonstrated somewhat better performance in identifying patients suitable for ABI screening. Deep learning (DL) models utilizing multiple neural networks capable of accounting for the sequence and timing of clinical events in a patient’s history can achieve superior discrimination performance in identifying those who should be screened for PAD. Compared to traditional risk score models and ML models, a deep learning model using time-series data on the entirety of the EHR built by Ghanzouri et al. achieved an average AUC of 0.96 for PAD detection [7]. Models like these offer the capability of using the vast EHR to summarize risk of PAD in complex patients, which can improve the efficiency in identifying who exactly should be screened with ABI testing.

#### Imaging Data

Imaging modalities have shown promise in aiding in the diagnosis of PAD by evaluating anatomic characteristics. However, the analysis of these data is complicated by variations in imaging interpretation. Specifically, point-of-care duplex ultrasound (DUS) arterial spectral Doppler data have often been challenging to interpret due to wide inter-observer variations. As an alternative, Normahani and colleagues introduced the idea of using ML models to analyze DUS to aid the diagnosis of PAD [8]. In a study of 305 patients with diabetes mellitus from which 590 waveform images were sampled at the ankle vessels, investigators reconstructed waveform signals and extracted time and time–frequency domain features including peak value and signal to noise ratio [8]. DL models using long short-term memory networks (LSTM) were then used to classify raw signals as PAD or no-PAD, while logistic regression (LR) and support vector machines (SVM) were used for classification of extracted features [8]. Their final model used a combination of DL and LR modeling and achieved an accuracy of 88% and AUC of 0.93 in discriminating PAD status [8]. Additionally, this model offered the benefits of standardization and reduced variation in data interpretation compared to point-of-care DUS [8]. Luo and colleagues have automated waveform classification of lower extremity arterial doppler (LEAD) ultrasound studies with the assistance of ML models [9]. They aimed to detect disease presence while assessing PAD in multiple levels: aortoiliac disease vs femoropopliteal disease vs tibial vessel disease and achieved accuracy of 88.2%, 90.1%, and 90.5%, respectively [9].

Contrast-enhanced magnetic resonance imaging (CE-MRI) is another imaging modality that can assess PAD status by analyzing the textural properties of skeletal calf muscles, as blood circulation and perfusion are impaired in PAD patients. Khagi and colleagues designed a ML model to extract textural features including homogeneity, contrast, and boundaries from CE-MRIs [10]. The study included 20 matched controls and 36 PAD patients, who were further subcategorized based on 6-min graded treadmill completion status and history of diabetes. To train their models they used SVM, LR and extreme gradient boosting (XGB) [9]. They found that an ensemble model that combined multiple algorithms and a technique to reduce the number of redundant features achieved 94% accuracy and an AUC of 0.94 when discerning PAD cases from controls compared to LR, SVM, and XGB alone, the latter of which achieved AUCs of 0.69, 0.79, and 0.9, respectively.

Recent imaging analyses have also focused on indirectly detecting early stages of PAD through identification of atherosclerosis. Mueller and colleagues leveraged the fact that subtle changes in vascular structures like those in the eyes are often representative of earlier stages of PAD [11]. They analyzed color fundus photography (CFP) images with deep attention-based Multiple Instance Learning (MIL) and developed a model that achieved 83.7% accuracy and an AUC of 0.89 when distinguishing patients with PAD from controls. Additionally, to minimize invasive data collection and improve cost efficiency, other imaging modalities have also been coupled with ML algorithms and have been found to be efficacious in aiding PAD diagnosis. Shahrbabak and colleagues developed DL models to analyze non-invasive brachial and tibial arterial pulse volume recordings (PVR) and arterial BP waveforms to detect and measure PAD severity [12]. They found that compared to ABI alone which achieved an AUC of ≤ 0.59, DL-enabled PVR waveform analysis and DL-enabled Arterial BP waveform analysis achieved AUCs of ≥ 0.89 and ≥ 0.96, respectively [12]. Thus, the addition of DL analysis to already collected minimally invasive imaging data can be another way to improve detection of PAD.

#### Proteomics Data

Biomarker data in addition to clinical risk factors offer an additional layer of assistance in assessing PAD functional severity and prognosis. ML analysis of data in patients undergoing invasive peripheral angiography who were enrolled in the Catheter Sampled Blood Archive in Cardiovascular Diseases (CASABLANCA) study has been shown to provide reliable scoring strategies for diagnosing obstructive PAD [13]. Scoring was based on a patient's history of hypertension as a clinical variable and 6 biomarkers: midkine, kidney injury molecule-1, interleukin-23, follicle-stimulating hormone, angiopoietin-1, and eotaxin-1 [13]. By dividing the scaled score into 5 categories, researchers correlated higher scores with increasing stenosis severity. Using this approach, ML models produced a cross-validated AUC of 0.84 for detecting PAD. Additionally, given a 4.3-year follow-up, elevated scores were noted to predict shorter times to revascularization, providing prognostic information for clinicians. While the above approach highlights advances in proteomics and personalized medicine, its generalizability may be limited as most medical centers are unlikely to routinely collect these specified biomarkers. With this in mind, Sonnenschein and colleagues ​focused on clinical variables and routinely available biomarkers including complete blood counts, electrolytes, iron studies, and lipid panels [14]. They employed a random forest (RF) model using these data to identify PAD patients with stable and unstable forms of disease, defined as Fontaine Class I/II and III/IV, respectively. They found that their ML-generated scores for PAD status significantly correlated with patient ABI measurements (R = -0.38, *p* = 0.007). The group argued that such a tool could help clinicians determine which patients should receive additional imaging, including invasive diagnostics and more intensive management of PAD.

#### Other Novel Data

Other novel data and ML approaches to PAD detection include work from Al-Ramini and colleagues. Al-Ramini et al. have used ML models to integrate laboratory-based gait biomechanics data for classifying PAD status since PAD affects muscle structure and function [15]. Their neural network ML model, utilizing gait variables, achieved an accuracy of 89% when tested on a retrospective dataset of 227 patients previously diagnosed with PAD and 43 healthy controls. The real-world application of this ML model in analyzing gait signatures could facilitate earlier identification of PAD in patients and monitor disease progression, especially if implemented in continuous monitoring devices.

A summary of studies mentioned above can be found in Table 1.

**Table 1:** Summary of recent machine learning models developed to screen and diagnose peripheral artery disease

Author	Goal	Optimal model	AUC
Sonderman et al. [6]	Estimation of the presence of PAD, as defined by ABI < 0.90, in patients without prior ABI testing in their electronic records	RF and LR	0.72
Ghanzouri et al. [7]	Classification of PAD using both classical machine learning and deep learning algorithms and electronic health record data	Deep Learning	0.96
Normahani et al. [8]	Utility of ML techniques for the diagnosis of PAD from Doppler arterial spectral waveforms sampled at the level of the ankle in patients with diabetes	LR	0.93
Luo et al. [9]	To recognize and differentiate LEAD and carotid duplex ultrasound signals and waveforms and for the decisions regarding classifying the level and severity of atherosclerotic disease with AI techniques	Carotid – Random Forest LEAD – Hierarchical neural network	NR
Khagi et al. [10]	To detect the heterogeneity in the muscle pattern among PAD patients and matched controls using calf muscles contrast-enhanced MRI (CE-MRI) scans	Maximum Relevance Minimum Redundancy + RF	0.94
Mueller et al. [11]	To capture retinal imaging biomarkers on CFP images	Deep attention-based Multiple Instance Learning (MIL) model	0.89
Shahrbabak et al. [12]	To investigate the feasibility of PAD diagnosis based on the analysis of non-invasive arterial pulse waveforms called pulse volume recording (PVR) signals	Deep Learning	0.90
McCarthy et al. [13]	To identify clinical and biomarker predictors of clinically significant PAD in an at‐risk population of subjects enrolled in the Catheter Sampled Blood Archive in Cardiovascular Diseases study undergoing peripheral angiography	LASSO + LR	0.85
Sonnenschein et al. [14]	To establish a general workflow to identify discriminative multi-dimensional markers for potential clinical diagnostics of vascular intervention	RF	NR
Al-Ramini et al. [15]	ML models on gait features to distinguish individuals with PAD from healthy older individuals without PAD	NN and RF	NR

*CNN* convolutional neural network; *LASSO* least absolute shrinkage and selection operator; *LEAD* lower extremity arterial duplex; *LR* logistic regression; *NN* neural network; *NR* not reported; *PAD* peripheral artery disease; *RF* random forest

### Risk Stratification

While clinical symptoms may only manifest in the lower extremities, PAD can be a marker of systemic disease and has been proven to be a predictor of coronary artery disease severity [16]. Furthermore, the diagnosis of PAD confers a greater long-term risk of overall cardiovascular mortality than myocardial infarction, resulting in some groups calling for PAD to be considered a coronary heart disease equivalent [17]. Thus, developing models that can predict severity of disease in individual PAD patients can help clinicians identify which patients may need higher intensity care.

#### MACCE

Ross and colleagues used ML to analyze both structured and unstructured EHR-data from patients with a diagnosis of PAD at two tertiary medical centers to predict patients at high risk of MACCE at the time of diagnosis [18]. Specifically, using patients with PAD who went on to develop MACCE after diagnosis as positive cases and those who did not have MACCE as negative cases, they were able to develop a predictive model using data only from the time the patient entered the health system to the time of PAD diagnosis [18]. This approach would allow clinicians to identify prognosis at the time of PAD diagnosis. The group’s best performing model, a RF model, utilized 957 variables and achieved an AUC of 0.81. Subsequent sensitivity analysis of the model revealed that unstructured text data and International Classification of Disease/Current Procedural Terminology coding data significantly contributed to the accuracy of the model. Earlier identification of these high-risk patients could allow for more targeted and aggressive medical intervention [17].

#### MALE

Amputation rates are of special interest in the management of PAD and can be considered a component of major adverse limb events (MALE). Austin et al. evaluated the performance of different ML models in the analysis of clinical and socioeconomic data in a cohort of 88,898 Medicare patients with newly diagnosed PAD and diabetes mellitus to identify variables most associated with risk of lower extremity amputation and mortality at 1-year [19]. An RF model significantly outperformed LR in terms of model prediction error rate for each outcome (30% v. 65%). This group did not report an AUC for the RF model and the goal was largely to demonstrate the advantages of an ML model like RF over traditional LR models in prediction tasks.

Tsarapatsani used a large cohort of hypertensive patients in the German epidemiologic trial on ankle-brachial index (getABI) to predict the need for revascularization and/or amputation at 7-years using only 12 clinical and laboratory features [20]. Their optimal model, an RF model, achieved an AUC of 0.73. As such this model might best be used in the primary care setting in identifying hypertensive patients who have the highest risk of long-term MALE for more aggressive medical management of modifiable risk factors or earlier referral to vascular specialists for treatment as soon as symptoms arise.

Using data from 255 patients with intermittent claudication, Rhavindran and colleagues trained a LASSO model to predict the risk of PAD's progression to critical limb threatening ischemia (CLTI) at 2- and 5-years, MACE at 5-years, MALE at 5 years, and the risk of 2 or more revascularization procedures within 5 years [21]. Given the small sample size they used bootstrapping to resample observations from a single data set, expanding their sample to 10,000 patients. Their prediction model produced AUCs for individualized outcomes ranging from 0.83–0.89. Of note, their models uniquely incorporated compliance with initial treatment strategy (best medical therapy, supervised exercise therapy, and endovascular intervention) as a feature of the LASSO model.

Given the proclivity for patients with PAD to undergo endovascular or open surgical interventions, there have been a variety of models developed to predict outcomes following procedures. Cox et al. utilized the American College of Surgeons National Surgical Quality Improvement Program (NSQIP) database to identify a cohort of 14,444 cases of patients undergoing lower extremity endovascular intervention for PAD [22]. Using the data from this cohort they developed an RF model to predict 30-day amputation rates following endovascular interventions, achieving an AUC of 0.81. Liu and colleagues performed a retrospective study using random survival forest (RSF) prediction model to predict rates of amputation-free survival (AFS) following successful inpatient revascularization for PAD [23]. The optimal model achieved AUCs of 0.82, 0.8, and 0.79 for predicting AFS at 1-, 3-, and 5-years post-revascularization, respectively [23]. This group compared their model to the GermanVasc score, a previously published ML model that predicts AFS at 5-years and found that their model outperformed the GermanVasc (c-index 0.78 v. 0.73, respectively) [24]. A limitation of this study, however, was sample size (*n* = 1260) and data source (single institution), which may hinder generalizability as institutional practice patterns may have undue influence on the model [24].

More recently, Li et al. used the Society for Vascular Surgery Vascular Quality Initiative (VQI) database to publish a series of papers predicting MALE and mortality at 1-year in patients undergoing infrainguinal bypass, suprainguinal bypass, and endovascular therapy (EVT) for PAD [25–27]. These studies are unique in several ways. They compared the performance of several models using preoperative features and found the XGB algorithm to have the most robust performance across multiple outcome prediction models used to predict 1-year MALE after infrainguinal bypass (AUC 0.94), suprainguinal bypass (AUC 0.92), and EVT (AUC 0.94). Additionally, following identification of XGB as the optimal algorithm in predicting the primary outcome using preoperative features, additional models were made incorporating intra-operative (procedural) features and post-operative features in an additive stepwise approach. The addition of intra-operative data to the models did not significantly alter performance, while the addition of post-operative features improved performance, resulting in AUCs of 0.96, 0.98, and 0.98, for patients undergoing infrainguinal bypass, suprainguinal bypass, and EVT, respectively. The group argued that the creation of models at each phase of a patient’s care would allow for maximum opportunity to mitigate adverse events and even offered a clinical workflow decision matrix utilizing the model output at each phase of care to guide decision-making. They provided a framework on the best way to implement these models clinically, suggesting using the pre-op model to identify high risk patients for interventions and consideration of further optimization or a different surgical approach. For example, for patients identified as high-risk for 1-year MALE or mortality via the intraoperative prediction model they suggested admission to higher levels of care for closer monitoring of these patients. Strategies for close follow-up, multidisciplinary post-operative care and rehabilitation were suggested for patients identified as high-risk by the postoperative model. Furthermore, this group made their model code publicly available via GitHub with suggestions for implementation at other centers participating in VQI and the staff required to do so. Finally, these models also maintained performance with respect to age, race, ethnicity, and rurality subgroups, complying with calls to develop models that demonstrate “fairness” [28].

The use of biomarkers in ML have also been shown to be useful in MALE prediction. Li and colleagues recruited a cohort of PAD and non-PAD patients and then performed propensity-matching to achieve a final cohort of 270 patients for model development [29]. They collected clinical data and levels of 37 proteins at the onset of the study, identifying 6 that were differentially expressed between the matched cohorts and then observed the groups for MALE over 2 years. They then used these 6 biomarkers as input features to train a RF model, achieving 2-year MALE prediction AUC of 0.86. They then recruited an additional cohort of 189 patients with PAD and 88 patients without PAD for prospective model validation. With this validation cohort, the model achieved an AUC of 0.84 for predicting 2-year MALE. Like their previous studies, this group discussed real-world application of their results, suggesting use of these biomarkers as a way for general practitioners to screen for PAD, then following confirmation of diagnosis by ABI, use of the model to identify those at high-risk for 2-year MALE.

#### Short Term Mortality, Readmission, Acute Renal Failure

Zhang and colleagues used the National Inpatient Sample database (NIS) to predict risk of in-hospital mortality in patients admitted with a primary diagnosis of PAD [30]. They used in-hospital data from a cohort of 150,921 patients and developed four ML models using RF, XGB, light gradient boosting, and LR algorithms. The XGB model demonstrated the highest AUC of 0.85, though performance across all models evaluated did not differ significantly with AUCs ranging from 0.83–0.85 [30]. The three most critical features for model performance were the number of diagnoses, procedures accrued during the hospitalization, and patient age. Cox et al. using the previously discussed NSQIP cohort of patients undergoing lower extremity endovascular intervention for PAD developed RF models to predict 30-day procedure mortality, as well as 30-day unplanned readmission [31]. AUC for 30-day mortality was 0.75 while the model for 30-day unplanned readmission demonstrated lower performance with AUC of 0.68 [31]. Using the same data and citing the commonality of contrast induced nephropathy following endovascular interventions, they also developed an RF model to predict 30-day acute renal failure in patients with AUC of 0.81 [32].

### Advanced Phenotyping

#### Unsupervised Learning

Unsupervised learning is a subclass of ML that uses unlabeled data to find patterns across data points and as such can be used to identify patients with shared prognoses. Zhang and colleagues used an unsupervised ML algorithm, specifically k-means clustering, to identify two novel clusters of patients with lower extremity PAD in a cohort of 460 patients based on baseline patient clinical characteristics at initial visit for PAD [33]. Patients in cluster 1 were noted to have significantly higher age, body mass index (BMI), neutrophil levels, prevalence of smoking, hypertension, and diabetes. Subsequent analysis of PAD specific 1-year outcomes showed that patients in cluster 1 had significantly lower ABIs, more severe claudication symptoms, higher Rutherford classification, and higher rates of tissue loss and amputation. Thus, the authors distinguished the two clusters as severe (cluster 1) and mild (cluster 2) PAD subtypes. The baseline variables identified as significantly different between the two groups were then used to develop a predictive model to identify patients belonging to the severe subtype with a reported AUC of 0.76. Such a model could be used to prospectively guide patient care, depending on what cluster a patient is most closely related to on initial evaluation.

Left untreated PAD can progress to chronic limb threatening ischemia (CLTI). Using one of the more novel applications of cluster modeling, McGinigle and colleagues used their institutional data for patients undergoing open and/or endovascular treatment of CLTI to develop a classification system for 1-year mortality, akin to tumor, node, metastasis (TNM) staging in cancer patients [34]. To accomplish this, the team abstracted patient demographics, presence and severity of comorbidities, Wound Ischemia foot Infection (WIfI) scores, and anatomic patterns of PAD from patient charts via manual chart review and natural language processing. These data were subsequently used in a supervised latent Dirichlet allocation (sLDA) topic model algorithm to cluster patients into “stages'' based on rates of 1-year mortality. Stage I, II, and III corresponded to 1-year mortality rates of 7.6%, 13.8%, and 18.9%. The authors argued that this robust staging system could help clinicians and patients develop a more patient-centered approach to CLTI management. However, generalizability of this model is limited by the sample size (*n* = 285), its retrospective nature, and single institution origin. The same group performed a subsequent study to develop a supervised clustering model using data from the Project of Ex Vivo Vein Graft Engineering via Transfection (PREVENT) III clinical trial [35]. Using the primary composite outcome of 1-year CLTI-free survival, their model was able to sort patients undergoing infrainguinal bypass into 3 distinct stages with decreasing rates of CLTI-free survival (82.3% v. 61.1% v. 53.4%). However, it is important to note that this group was not able to validate this cluster model in a real-world cohort of patients and acknowledged that external validation would be required to confirm their findings.

A summary of the above studies is detailed in Table 2.

**Table 2:** Summary of recent machine learning models used in peripheral artery disease risk stratification

Author	Goal	Optimal model	AUC
Ross et al. [18]	Prediction of MACCE after PAD diagnosis	RF	0.81
Austin et al. [19]	1-year Lower-Extremity Amputation Risk	RF	NR
Tsarapatsani et al. [20]	Predict need for amputation and/or revascularization in patients with hypertension	RF	0.73
Rhavindran et al. [21]	Predict risk of progression to CLTI at 2- and 5-years, MACE at 5-years, MALE at 5-years, and risk of 2 + revascularization procedures in 2 years	LASSO	0.83–0.89
Cox et al. [22]	Predict 30-day amputation risk after endovascular intervention	RF	0.81
Liu et al. [23]	Predict rates of amputation-free survival following successful inpatient revascularization at 1, 3, and 5 years	Random Survival Forest	0.82 (1-year), 0.8 (3-year), 0.79 (5-year)
Li et al. [25]	Prediction of 1-year MALE in patients undergoing endovascular intervention	XGB	0.94 – 0.98
Li et al. [26]	Prediction of 1-year MALE in patients undergoing infrainguinal bypass	XGB	0.94 – 0.96
Li et al. [27]	Prediction of 1-year MALE in patients undergoing suprainguinal bypass	XGB	0.92 – 0.98
Li et al. [29]	Prediction of 2-year MALE in patients with PAD	RF	0.84
Zhang D et al. [30]	Prediction of in-hospital mortality in patients admitted for PAD	XGB	0.85
Cox et al. [31]	Prediction of 30-day procedure-related mortality and 30-day unplanned readmission following endovascular intervention	RF	0.75 (Mortality) 0.68 (Unplanned readmission)
Cox et al. [32]	Prediction of 30-day acute renal failure after endovascular intervention	RF	0.81
Zhang et al. [33]	Creation of a PAD subtype using unsupervised learning and neutrophil-related biomarkers	K-means clustering	NA
McGinigle et al. [34]	Create TNM-like staging for patients with CLTI based on risk of 1-year mortality using unsupervised learning	sLDA	NA
Chung et al. [35]	Use topic model cluster analysis to risk stratify patients with CLTI by 1-year CLTI-free survival	sLDA	NA

*CLTI* chronic limb threatening ischemia; *LASSO* least absolute shrinkage and selection operator; *NA* not applicable; *NR* not reported; *PAD* peripheral artery disease; *RF* random forest; *sLDA* supervised latent Dirichlet allocation; *XGB* extreme gradient boosting

## Conclusions

Our review highlights the potential of ML to serve as a valuable tool for supporting physicians in their clinical decision-making. Current research shows that ML models could allow for more effective screening and earlier diagnosis of PAD using data types such as the EHR, imaging, and proteomics. ML models can also improve risk stratification. Models developed using algorithms such as LASSO, XGB and RF have the potential to assist physicians in accurately predicting the progression of PAD or risk of MACCE across diverse patient groups. Utilization of such models early in the diagnosis process could allow for better management of comorbidities prior to PAD treatment.

While ML has the capability to more accurately and efficiently diagnose and classify PAD, therefore potentially improving patient outcomes while reducing cost of care, its use currently has limitations. Some models could be difficult to implement in everyday practice such as those using proteomics data, and additional research is needed to improve ML generalizability. Additional studies will be required to implement such ML models in clinical workflows and demonstrate their usefulness in patient care. Regardless of the model used or how it is used, AI and ML-based PAD care will likely still require physician or human oversight to prevent potential errors in the near term.

Overall, ML/AI has the potential to revolutionize PAD diagnosis, prognosis and treatment, however, further studies are essential to address current limitations and successfully implement these tools into clinical practice.

## Key References

- Ghanzouri, I., Amal, S., Ho, V. et al. Performance and usability testing of an automated tool for detection of peripheral artery disease using electronic health records. Sci Rep 12, 13364 (2022). 10.1038/s41598-022-17180-5
- Cox M, Reid N, Panagides JC, Di Capua J, DeCarlo C, Dua A, Kalva S, Kalpathy-Cramer J, Daye D (2022) Interpretable Machine Learning for the Prediction of Amputation Risk Following Lower Extremity Infrainguinal Endovascular Interventions for Peripheral Arterial Disease. Cardiovasc Intervent Radiol 45:633–640
- McGinigle KL, Freeman NLB, Marston WA, Farber A, Conte MS, Kosorok MR, Kalbaugh CA (2021) Precision Medicine Enables More TNM-Like Staging in Patients With Chronic Limb Threatening Ischemia. Front Cardiovasc Med 8:709904

## Data Availability

No datasets were generated or analysed during the current study.

## Declarations

### Disclaimer

The views expressed in this material are those of the authors, and do not reflect the official policy or position of the U.S. Government, the Department of Defense, the Department of the Navy, or Naval Medical Center San Diego.
